# 

**Published:** 1892-11

**Authors:** 


### TABLE OF CONTENTS.

ORIGINAL COMMUNICATIONS. PAGE

Dental Law and Dental Education. By W. E. MAGILL, D.D.S., Erie, Pa. 785

Crown- and Bridge-Work. (*Continued.*) By DR. C. M. RICHMOND, New York . . . . . . . . . . . . . . . . . . . . . . . . . . . . . . 791

The Philosophy of the Homœopathic Law of Cure, and the Advantage to the Dentist of a Correct Knowledge of its Application. By CHARLES H. TAFT, D.M.D., Cambridge, Mass. . . . . . . . . . . . . . . . . 793

The Value of Illustrations in the Lecture-Room. By L. D. McINTOSH, M.D., D.D.S. . . . . . . . . . . . . . . . . . . . . . . . . . . . . 801

How can we Magnify the Importance of our Calling? By G. A. MILLS, New York City . . . . . . . . . . . . . . . . . . . . . . . . . . 806

Anti-Conservative Treatment of Exposed Dental Pulp. By CHARLES HARKER, D.D.S. . . . . . . . . . . . . . . . . . . . . . . . . . . 815

A Case. By CHARLES B. ATKINSON, D.D.S., New York . . . . . . . . 822

Hints Worth Remembering. By GEORGE A. MAXFIELD, D.D.S. . . . . 825

REPORTS OF SOCIETY MEETINGS.

American Dental Association . . . . . . . . . . . . . . . . . . . . . . . 825

EDITORIAL.

Essays for the Columbian Congress . . . . . . . . . . . . . . . . . . 837

BIBLIOGRAPHY . . . . . . . . . . . . . . . . . . . . . . . . . . . . . . . 839

FOREIGN CORRESPONDENCE.

Response to Drs. Heitzmann and Taft . . . . . . . . . . . . . . . . . 840

OBITUARY.

American Dental Association . . . . . . . . . . . . . . . . . . . . . . . 844

CURRENT NEWS.

Committee on Essays (No. 17) of the World's Columbian Dental Congress . 846

Recent Patents . . . . . . . . . . . . . . . . . . . . . . . . . . . . . . 847

Board of Examiners of District of Columbia . . . . . . . . . . . . . . 848

World's Columbian Dental Congress Officers elected at Chicago . . . . . 848

